# Photoactive Zr-aromatic hybrid thin films made by molecular layer deposition

**DOI:** 10.1039/d2ra02004a

**Published:** 2022-05-25

**Authors:** Melania Rogowska, Ellen Bruzell, Håkon Valen, Ola Nilsen

**Affiliations:** Centre for Materials Science and Nanotechnology, Department of Chemistry, University of Oslo 0315 Oslo Norway ola.nilsen@kjemi.uio.no melaniar@smn.uio.no; Nordic Institute of Dental Materials 0855 Oslo Norway

## Abstract

The principle of antimicrobial photodynamic therapy (PDT) is appealing because it can be controlled by an external light source and possibly the use of durable materials. However, to utilise such surfaces requires a process for their production that allows for coating on even complex geometries. We have therefore explored the ability of the emerging molecular layer deposition (MLD) technique to produce and tune PDT active materials. This study demonstrates how the type of aromatic ligand influences the optical and antimicrobial properties of photoactive Zr-organic hybrid thin films made by MLD. The three aromatic dicarboxylic acids: 2,5-dihydroxy-1,4-benzenedicarboxylic acid, 2-amino-1,4-benzenedicarboxylic acid and 2,6-naphthalenedicarboxylic acid have been combined with ZrCl_4_ to produce hybrid coatings. The first system has not been previously described by MLD and is therefore more thoroughly investigated using *in situ* quartz crystal microbalance (QCM), Fourier transform infrared (FTIR) and UV-Vis spectroscopy. The antibacterial phototoxic effects of Zr-organic hybrids have been explored in the *Staphylococcus aureus* bacteria model using a UVA/blue light source. Films based on the 2,6-naphthalenedicarboxylic acid linker significantly reduced the number of viable bacteria by 99.9%, while no apparent activity was observed for the two other photoactive systems. Our work thus provides evidence that the MLD technique is a suitable tool to produce high-quality novel materials for possible applications in antimicrobial PDT, however it requires a careful selection of aromatic ligands used to construct photoactive materials.

## Abstract 2

Photoactive organic–inorganic hybrid thin films for potential use in antimicrobial photodynamic therapy (PDT) were fabricated based on Zr clusters and three different aromatic dicarboxylic acid linkers using the molecular layer deposition (MLD) technique.

## Graphical abstract

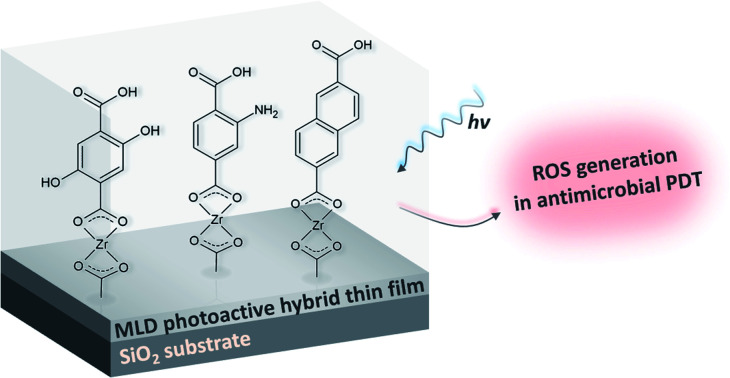

## Introduction

An increasing range of the functional materials used in our daily life are based on interactions with optical radiation, both from a practical and sustainable approach. Photoactive materials and compounds are used in optoelectronic devices such as light-emitting diodes and solar cells, sensors, photocatalysts, and in biological systems.^1^ One clinically established light-induced method is photodynamic therapy (PDT). This method utilises compounds called photosensitisers that, in the presence of oxygen, are exposed to optical radiation of wavelengths matching the absorption spectrum of the target molecule. Following excitation, photochemical reactions take place generating reactive oxygen species (ROS) that can damage biomolecules. PDT is approved for skin pre-cancer and cancer treatments, and is also used as a diagnostic tool.^2^ Moreover, this therapeutic procedure is applied clinically and experimentally to kill or damage Gram-positive and Gram-negative bacteria, viruses, fungi and parasites, in particular for dental peri-implant infections.^3,4^ Light-induced antimicrobial treatment could also be applied for decontamination and disinfection of various environmental surfaces to reduce risk of infection and transmission of pathogens.^5^

Photoactive materials and surfaces containing immobilised photosensitizers can be designed with ultra-high precision on the atomic/molecular level through the atomic/molecular layer deposition (ALD/MLD) technique. ALD is an established method for coating substrates with high-quality uniform and conformal inorganic materials for microelectronics, photovoltaics, electroluminescent displays, among others.^6^ It is based on sequential self-limiting gas–surface reactions that enables precise thickness and composition control of the thin film. The MLD method expands on the ALD approach by allowing growth with larger building blocks to produce entirely organic or hybrid organic–inorganic films.^7^

Aromatic dicarboxylic acids are popular as organic linkers in MLD due to their rigid structure and high reactivity. In addition, they provide interesting optical properties such as absorption in the UV and visible light region.^8–11^ Furthermore, by combining various organic and inorganic components we can design the optical absorption and luminescence of such hybrid materials. We recently reported how the type of transition metal affects the optical properties of MLD hybrid materials based on 2,6-naphthalenedicarboxylic acid as the linker.^12^ For instance, the presence of d^0^-metals such as Ti, Zr or Hf shifts the light absorption towards the visible part of the spectrum and increases the potential application of such hybrid materials as photoactive agents. Moreover, the light absorption properties can be further tuned by increasing the size of the aromatic backbone or by adding various functional groups.^13,14^

In this work, we demonstrate a successful MLD growth of a novel photoactive hybrid thin film material based on surface reactions of zirconium (ZrCl_4_) and 2,5-dihydroxy-1,4-benzenedicarboxylic acid (abbreviated here as 25D-BDC). The 25D-BDC aromatic linker has high near-UV absorption, which can be beneficial for application in certain types of antimicrobial PDT where shorter wavelengths are needed. Zirconium, on the other hand, is a non-toxic biocompatible element,^15^ while zirconium dioxide (ZrO_2_) thin films fabricated by ALD are shown to promote cell adhesion and spreading, but also some antibacterial activity.^16^

We further explored the antibacterial properties of our photoactive hybrid films based on Zr and three different aromatic dicarboxylic acids, *i.e.* the 25D-BDC linker described above, 2-amino-1,4-benzenedicarboxylic acid (2A-BDC) and 2,6-naphthalenedicarboxylic acid (26-NDC). The experiments were performed *in vitro* using *Staphylococcus aureus* as a model organism. This Gram-positive bacterium is a major human pathogen and a leading cause of healthcare-associated infections.^17^*S. aureus* infections may be challenging to treat due to biofilm formation, persister cells formation and antibiotic resistance, such as the methicillin-resistant *S. aureus* (MRSA).^18^ PDT has been reported to have appreciable killing effectiveness on drug-resistant bacteria and claimed to be associated with a potential low risk of drug resistance development.^19^ We foresee that uniform, photoactive antimicrobial coatings prepared by the MLD technique could be applied in PDT on small medical devices for external use and/or surgical instruments to prevent and treat bacterial infections such as those caused by the *S. aureus* bacteria. Furthermore, in the current work we show how the type of organic linker affects the light absorption properties of Zr-organic hybrid materials and thus their antimicrobial phototoxicity.

## Experimental methods

### Molecular layer deposition

All the Zr-hybrid thin films were grown using an F-120 Sat ALD reactor (ASM Microchemistry) at a reactor pressure of 6.6 mbar. Nitrogen (99.999%, Schmidlin Sirocco 5 V1 N_2_ generator) was used both as carrier and purging gas with a total flow of *ca*. 400 sccm (cm^3^ min^−1^) throughout the experiments (inner chamber flow 300 sccm and outer chamber flow 100 sccm). The precursors used in this study were ZrCl_4_ (Strem Chemicals, >99.9%), 2,5-dihydroxy-1,4-benzenedicarboxylic acid (25D-BDC) (Sigma-Aldrich, 98%), 2-amino-1,4-benzenedicarboxylic acid (2A-BDC) (Sigma-Aldrich, 99%) and 2,6-naphthalenedicarboxylic acid (26-NDC) (Sigma-Aldrich, 99%). The sublimation temperatures used were 165 °C for ZrCl_4_ and 225 °C for 25D-BDC, 2A-BDC and 26-NDC. The thin films were deposited on Si (100) substrates precleaned with ethanol (VWR, 99.96%). The growth rate of the Zr-25D-BDC film was measured as a function of deposition temperature and number of cycles.

### Characterization of the films

*In situ* quartz crystal microbalance (QCM) analysis of the Zr-25D-BDC system was conducted using two 6 MHz gold-coated quartz crystals (Inficon) placed *ca*. 5 cm apart along the flow direction using a homemade holder. The crystals were used to monitor the mass increase during the deposition and to optimize the precursor pulse and purge lengths. The signal was recorded using a Colnatec Eon-LT unit and processed by averaging over 16 consecutive cycles. The temperature was stabilized for 2 h before the experiments began to minimize any effects of fluctuating temperatures. The thickness and refractive index of the films were determined with an alpha-SE spectroscopic ellipsometer (J. A. Woollam) in the wavelength range of 390–900 nm at an incident angle of 70°. The data were fitted to a Cauchy model using the CompleteEASE software. The thickness of the native oxide layer was determined before the deposition process and added as an individual layer in the interpretation of film thickness. Fourier transform infrared spectroscopy (FTIR) measurements of the Zr-25D-BDC film were performed using a Bruker Vertex 70 spectrometer equipped with a Pike VeeMAX III specular reflection accessory with an angle of incidence at 75°. Data were acquired in the reflection mode using an average of 64 scans in the wavenumber range of 4000–370 cm^−1^ and 4 cm^−1^ resolution. Films for FTIR measurements were deposited on electropolished steel substrates. A spectrum obtained from the uncoated electropolished steel substrate was used as the background during the measurements. FTIR measurement of the organic compound was performed using the same instrument in the transmission mode. The pellet was prepared by mixing approximately 1 mg of the sample with 100 mg KBr and pressed into a pellet in a hydraulic press. UV-Vis transmittance spectra of the films were collected with a Shimadzu UV-3600 UV-Vis-NIR spectrophotometer in the wavelength range of 200–850 nm with 1 nm resolution. Water contact angle measurements were performed using a Theta Lite optical tensiometer (Biolin Scientific). For each measurement, a 4 μl droplet of distilled water was administered by a syringe and placed on the surface of the film. The contact angle was measured for 60 s at room temperature.

### Antibacterial phototoxicity experiments

#### Light source

The irradiation chamber (Polylux PT, Dreve) was equipped with two compact fluorescent tubes emitting in the UVA (Ralutec 9W/78, Radium) placed on each side of one blue light tube emitting blue light (Osram 9W/71). The combined emission wavelength range was 350–500 nm, with the maximum emission at around 368 nm (UVA) and 447 nm (blue) (irradiation chamber configuration: UVA-blue-UVA). Emission spectra were determined with a calibrated double monochromator spectroradiometer (model DTM300, Bentham Instruments Ltd) and the irradiance was monitored with regular intervals with a UDT 271 radiometer (United Detector Technology) calibrated towards the spectroradiometer. The radiometer was equipped with probes sensitive in the blue (268BLU S/N 23476, calibrated at 450 nm) and UVA (268UVA S7N 8U021, calibrated at 365 nm) parts of the spectrum. The average irradiance of the light tubes (stable after 20 min) was 9 mW cm^−2^ (±5%).

#### Phototoxicity on planktonic bacteria

Stock cultures of *Staphylococcus aureus* (strain Newman) were stored at −80 °C in brain heart infusion (BHI, CM1135, Oxoid Ltd) supplemented with 30% glycerol. The fresh cultures were prepared by growing *S. aureus* overnight (ON) for 18 h in BHI in an incubator (Panasonic MCO-19M) at 37 °C and 100% humidity under normal atmospheric conditions supplemented with 5% CO_2_. For ON culture preparation, the sterile inoculating loop was dipped in the stock cultures and transferred into 10 ml BHI. The ON cultures were centrifuged at 5000 g for 5 min and resuspended in 0.9% saline solution (NaCl, Sigma-Aldrich, >99%) to an optical density at 600 nm (OD_600_) of approximately 1.0 using a spectrophotometer (Thermo Scientific Spectronic 200E spectrometer). OD_600_ of 1.0 corresponds to about 10^8^ colony forming units (CFU) ml^−1^. The bacteria suspension was then diluted 1 : 10 in 0.9% NaCl to achieve a desired bacteria concentration of approximately 10^7^ CFU ml^−1^. Thin films for antibacterial assays were deposited on microscope cover glasses (*ø* = 20 mm, VWR) precleaned with soap, water and ethanol followed by 8 min plasma treatment (PDC-002-CE plasma cleaner, Harrick Plasma). Films and uncoated cover glass controls were placed in two 12-well plates (Costar, Corning Inc.) and exposed to 1 ml of prepared bacteria suspension followed by 90 min incubation (37 °C, 100% humidity and 5% CO_2_). During the procedure, the samples were protected from light using aluminium foil. Half of the samples were placed in the irradiation chamber and irradiated with UVA/blue light for 20 min with a light dose of 11 J cm^−2^. A 30 min post-irradiation incubation time was applied to ensure completion of dark toxicity reactions following phototoxicity reactions. The samples were removed from the wells and transferred to 50 ml Falcon tubes containing 10 ml glass beads 3 mm in diameter (Merck) and 5 ml of 0.9% NaCl and vortexed for 30 s. The suspension was serial diluted in phosphate buffered saline (PBS, Bio-Whittaker, Lonza) and plated onto BHI agar plates (Agar bacteriological No. 1, Oxoid Ltd). The supernatant from the wells was also serial diluted and plated onto BHI agar plates. After approximately 24 h incubation (37 °C, 100% humidity and 5% CO_2_) the colonies were counted, and CFU ml^−1^ was calculated. For the Zr-26-NDC samples, three independent experiments were performed in triplicate (*n* = 9), whereas for the Zr-25D-BDC and the Zr-2A-BDC samples, two independent experiments were performed in triplicate (*n* = 6). To verify the inoculum concentration, the OD_600_ and the diluted 1 : 10 bacteria suspension were plated onto BHI agar plates during each experiment.

#### Phototoxicity on biofilm

The Zr-26-NDC samples were also tested against bacteria established as a 24 h biofilm. For biofilm assays, ON culture of *S. aureus* was diluted 1 : 100 in BHI. Films and uncoated cover glass controls were placed in two 12-well plates and exposed to 1.5 ml of prepared bacteria suspension followed by 24 h incubation (37 °C, 100% humidity and 5% CO_2_). The BHI was then removed from the wells and replaced by 1 ml of 0.9% NaCl. Half of the samples were placed in the irradiation chamber and irradiated with UVA/blue light for 20 min with a light dose of 11 J cm^−2^. After irradiation, the samples were placed back in the incubator for 30 min. The samples were removed from the wells and transferred to 50 ml Falcon tubes containing 10 ml glass beads 3 mm in diameter and 5 ml of 0.9% NaCl and vortexed for 30 s. The suspension was serial diluted in PBS and plated onto BHI agar plates. The experiment was performed in duplicate with three independent parallels (*n* = 6). The established biofilm was verified with a confocal laser scanning microscopy (CLSM, Olympus FluoView FV1200) using a diode laser emitting at 473 nm. The SYTO 9 staining (LIVE/DEAD™ Viability Kit, Thermo Fisher Scientific) was used to colour the biofilm on the surface. The scanning was performed using a 60 × water lens, 0.5 μm step size and a format of 512 × 512 pixels corresponding to an area of 88 × 88 μm.

The antibacterial properties were analysed using one-way analysis of variance (ANOVA) method and post-hoc *t*-test with Bonferroni correction. The significance level was set to *p* < 0.05. The bacterial reduction *R* was calculated in percent from treated sample (*A*) compared to untreated control (*B*):

1 %*R* = (*B*_CFU ml^−1^_ − *A*_CFU ml^−1^_)/*B*_CFU ml^−1^_ × 100

## Results and discussion

### Film growth

**Fig. 1:**
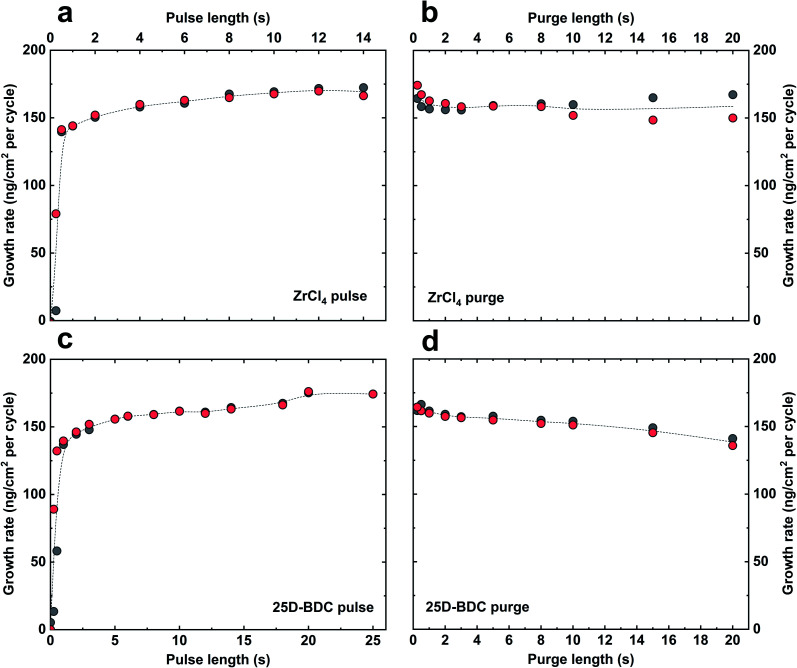
Test of self-saturating growth for the Zr-25D-BDC system. The growth rate as a function of pulse and purge lengths with a standard pulsing sequence of 4 s ZrCl_4_ pulse, 3 s N_2_ purge, 5 s 25D-BDC pulse, and 3 s N_2_ purge (4–3–5–3). The parameters that were varied are (a) ZrCl_4_ pulse length, (b) ZrCl_4_ purge length, (c) 25D-BDC pulse length and (d) 25D-BDC purge length. The deposition temperature was 260 °C. The grey circles represent the QCM sensor in the front of the reaction chamber and the red circles the QCM sensor in the back, situated *ca*. 5 cm apart. The dashed lines show the average value for two QCM sensors.

**Fig. 2:**
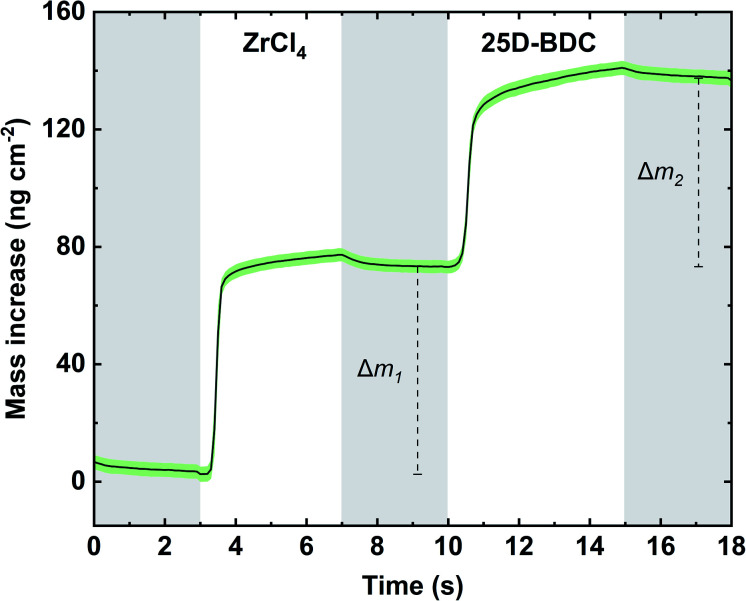
Mass increase as a function of time for Zr-25D-BDC system. ZrCl_4_ and 25D-BDC pulses were separated by inert gas (N_2_) purges (grey panels), with standard pulsing sequence of 4–3–5–3 s. The green shaded area represents the standard deviation for the QCM data (*n* = 16).

The growth dynamic and the saturation behaviour of the new Zr-25D-BDC system based on zirconium tetrachloride and 25D-BDC was initially investigated at 260 °C using an *in situ* QCM technique. Fig. 1 shows the growth rate as a function of inorganic and organic precursor pulse and purge length. The QCM experiment was performed by sequentially changing the pulse and purge length one at a time while keeping the other parameters constant at the standard pulsing sequence. The standard pulsing sequence used throughout the QCM experiment was 4 s ZrCl_4_ pulse, 3 s N_2_ purge, 5 s 25D-BDC pulse and 3 s N_2_ purge (termed here 4–3–5–3 s). We observed that for both organic and inorganic precursors, a 4–5 s pulse is sufficient to reach a complete saturation, and moreover, this experiment proves a self-saturating growth for both precursors. Fig. 2 shows the mass evolution during a MLD cycle sequence. The relative mass gain for each precursor sums to Δ*m*_1_(ZrCl_4_) : Δ*m*_2_(25D-BDC) = (70.71 ng cm^−2^/64.23 ng cm^−2^) = 1.10. This ratio is in good agreement with the calculated relative mass change from the possible ideal surface reactions during one MLD cycle with 1 : 1 stoichiometry for Zr and 25D-BDC, and where on average 2.3 of the Cl ligands on Zr reacts with the surface during the ZrCl_4_ pulse (149.55 g mol^−1^/135.79 g mol^−1^ = 1.10):

2 |−(OH)_2.3_ + ZrCl_4_ (g) = |−(O)_2.3_ZrCl_1.7_ + 2.3HCl (g)

Δ*m*_1_ = 149.55 g mol^−1^

3 |−(O)_2.3_ZrCl_1.7_ + 25D-BDC (g) = |−(O)_2.3_Zr(25D-BDC) + 1.7HCl (g)

Δ*m*_2_ = 135.79 g mol^−1^

**Fig. 3:**
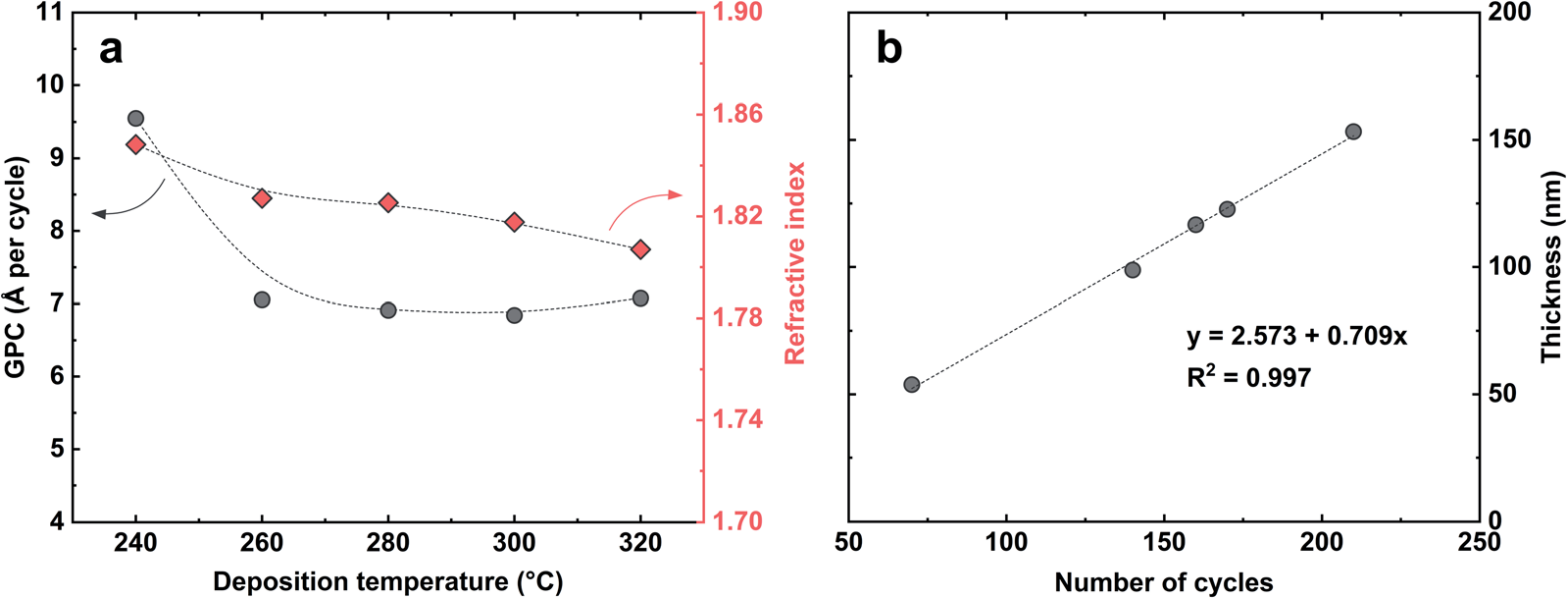
(a) The growth per cycle and the refractive index of the Zr-25D-BDC film as a function of the deposition temperature (*n* = 140). (b) The film thickness *versus* number of MLD cycles. In (b), the deposition temperature was 260 °C and the dashed line shows a fitted linear regression. The estimated uncertainties are within the sizes of the symbols.

The growth rate of the Zr-25D-BDC system was further studied in the temperature range of 240–320 °C using 140 MLD cycles and the standard pulsing sequence of 4–3–5–3 s, see Fig. 3a. The highest growth-per-cycle (GPC) value of 9.6 Å per cycle was observed at 240 °C. The GPC then decreases with increasing reaction temperature to 7.1 Å per cycle at 260 °C and remains nearly constant up to 320 °C showing an apparent MLD temperature window for this process between 260–320 °C. The refractive index slightly decreases with deposition temperature from 1.848 at 240 °C to 1.807 at 320 °C. Furthermore, the depositions within the entire studied temperature range yielded visually smooth and homogeneous thin films. For the rest of the experiments, we decided to fix the deposition temperature at 260 °C. Finally, we confirmed that the film thickness increases linearly with increasing number of deposition cycles as expected for an ideal ALD/MLD growth (Fig. 3b). The extrapolated graph in Fig. 3b intersects the *x*-axis at around 2.57 nm, supporting a near ideal growth without nucleation barriers. The Zr-25D-BDC film is amorphous as confirmed by X-ray diffraction (XRD) and exhibits low surface roughness as measured by atomic force microscopy (AFM) with the root-mean-square (RMS) roughness of 0.2 nm. The density of the film was estimated to be 2.04 g cm^−3^ from X-ray reflectivity (XRR) measurements.

The Zr-2A-BDC and Zr-26-NDC films were deposited as described in detail in our previous work.^11,12,20^ Specifically, the standard pulsing sequence for Zr-2A-BDC was 4–3–5–3 s and for Zr-26-NDC was 3–2–20–2 s. In both cases, the deposition temperature was set to 260 °C.

### Bonding characteristics

**Fig. 4:**
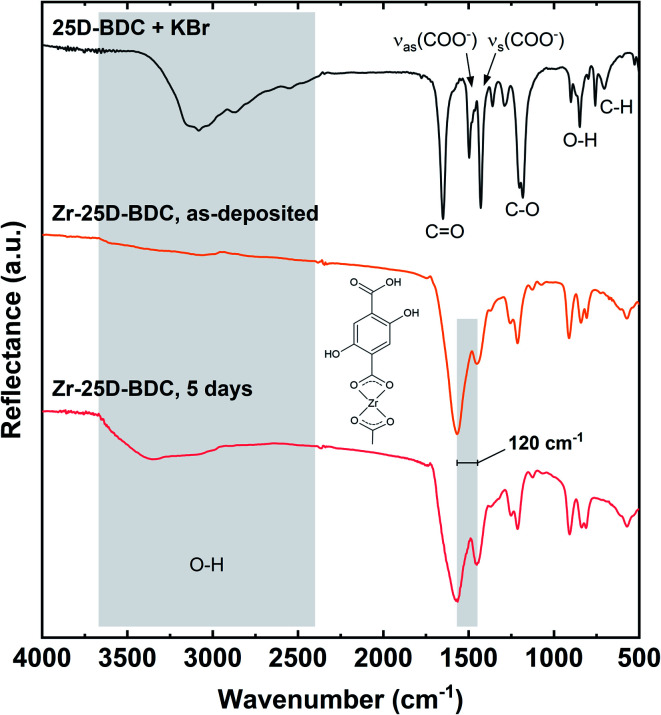
FTIR reflection spectra of representative hybrid thin film (123 nm) and the corresponding organic precursor. The insert shows the possible bonding mode for the Zr-25D-BDC system.

Fourier transform infrared spectroscopy (FTIR) was used to confirm the presence of the organic moiety in the deposited Zr-25D-BDC film and its coordination to the metal. In Fig. 4 we show FTIR reflection spectra of the as-deposited Zr-25D-BDC film and the same film after 5 days of air-storage at ambient atmosphere of 25 °C and around 70% humidity. For comparison, the spectrum of the corresponding carboxylic acid precursor is included as reference. The dominant absorption peaks seen at 1568 and 1448 cm^−1^ at the spectra of the films correspond, respectively, to asymmetric (ν_as_) and symmetric (ν_s_) stretching vibrations of the carboxylate group COO^−^.^8^ These peaks are slightly shifted towards the higher wavenumbers as compared to the pure organic precursor, which suggests a reaction with the metal component. The separation between asymmetric and symmetric stretching of the COO^−^ varies depending on how the carboxylate group coordinates to a metal atom. A splitting value, *Δ*, in the range between 50 and 150 cm^−1^ is typical for bidentate (chelating) complexes, monodentate complexes show a separation of *Δ* > 200 cm^−1^, and bridging complexes have *Δ* values between 130 and 200 cm^−1^.^21^ Given this information, the *Δ* = 120 cm^−1^ for Zr-25D-BDC film indicates a bidentate type of coordination, and a possible structure of this Zr-hybrid is shown in the inset of Fig. 4. A similar coordination mode was previously observed for the Zr-2A-BDC and Zr-26-NDC films.^12,20^

The absence of a sharp intense peak at around 1650 cm^−1^ from the C=O stretching vibrations of free COOH groups in the film proves that it coordinates to the zirconium atom during formation of the hybrid structure.^10^ Similarly, from the spectrum of as-deposited films, the absence of the broad absorption between 2400 and 3500 cm^−1^ with the maximum at around 3080 cm^−1^ arising from stretching vibrations of OH groups from COOH indicates that the oxygen is deprotonated and bonded to the metal.^22^ The films, however, absorb water into the structure as it can be seen as a broad band between 3670 and 2940 cm^−1^ in the spectrum of the film after 5 days of storage in ambient atmosphere. Simultaneously, the bands from the carboxylate group are broadened without any apparent changes in the coordination mode.

The spectra of the Zr-25D-BDC film also show the absorption at around 1210 cm^−1^, which corresponds to C–O stretching vibrations of carboxylic acid, the absorption at around 910 cm^−1^ is due to the out-of-plane bending vibrations of OH groups, and the double absorption peak at around 820 cm^−1^ is due to the out-of-plane bending vibrations of C–H bond in the aromatic ring of the 25D-BDC molecule.^23,24^ The broad character of the peaks from asymmetric and symmetric stretching vibrations of the carboxylate group can also suggest an amorphous character of the deposited films. Furthermore, no changes in the type of coordination mode nor possible decomposition of the films were observed in the FTIR spectra of the Zr-25D-BDC film deposited at different temperatures from 240 °C to 320 °C (data not shown).

### Photoabsorption characteristics

**Fig. 5:**
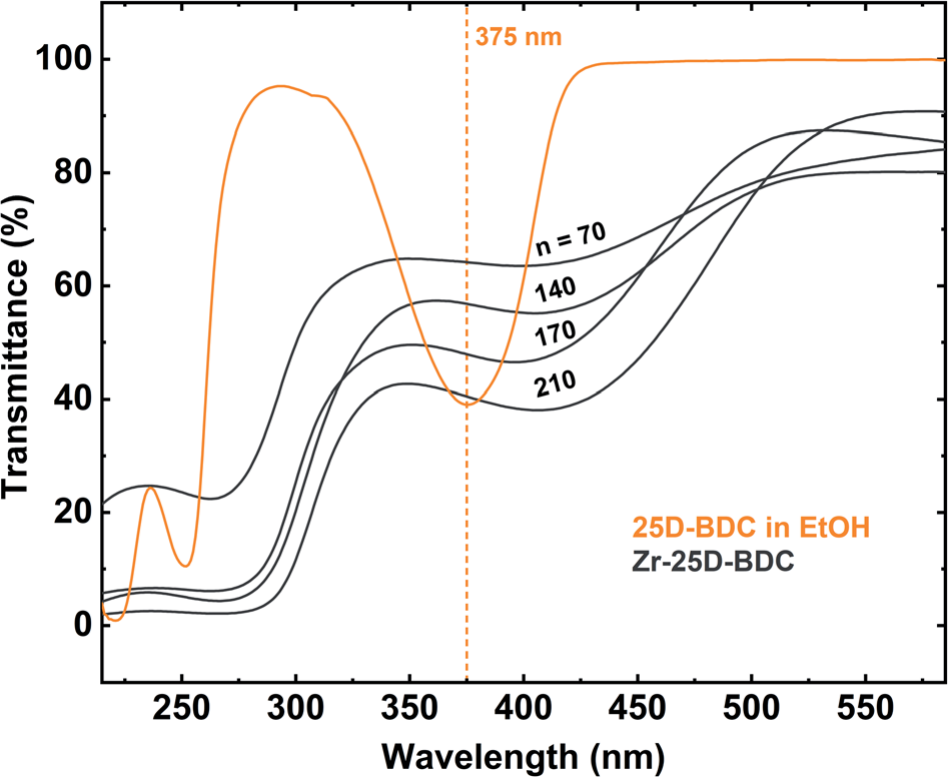
UV-Vis transmission spectra of the Zr-25D-BDC hybrid films with varying numbers of MLD cycles (*n*). The thickness of the films was 54 nm (*n* = 70), 99 nm (*n* = 140), 123 nm (*n* = 170) and 153 nm (*n* = 210). The orange line shows the spectrum of the corresponding organic precursor dissolved in ethanol with the maximum absorption peak at 375 nm.

The transmission spectra obtained by UV-Vis spectroscopy of the hybrid films with different thicknesses, corresponding to the number of MLD cycles (*n* = 70, 140, 170 and 210), and the solution spectrum of the 25D-BDC precursor are shown in Fig. 5. The strong band located at around 375 nm for 25D-BDC precursor is attributed to π–π* intraligand transition in the aromatic ring of the molecule. For the hybrid Zr-25D-BDC thin films the absorption peak has been redshifted toward the longer wavelengths with about 40 nm due to interactions between the organic ligand and a Zr atom. The films show strong absorption in the visible region with the absorption onset occurring below 500 nm and *λ*_max_ = 410 nm for thin film with *n* = 210.

**Fig. 6:**
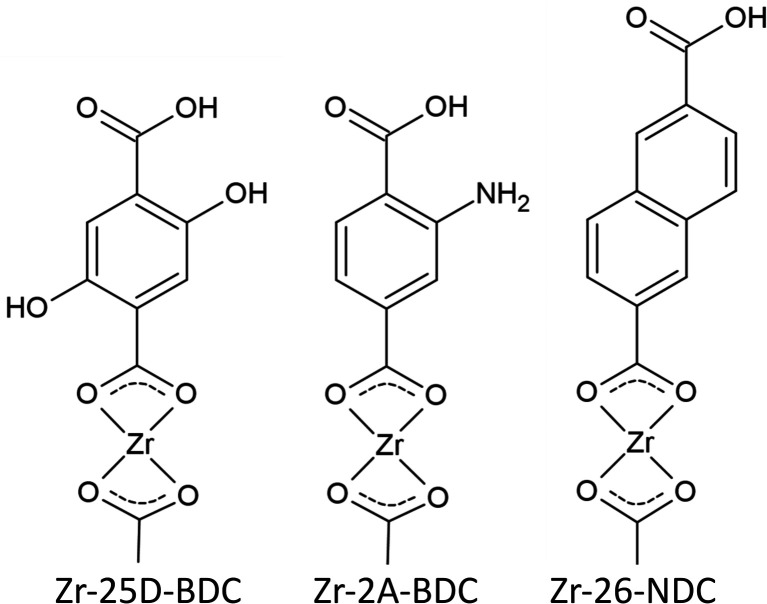
Possible structures of Zr-hybrids used in bacteria inactivation experiments.

**Fig. 7:**
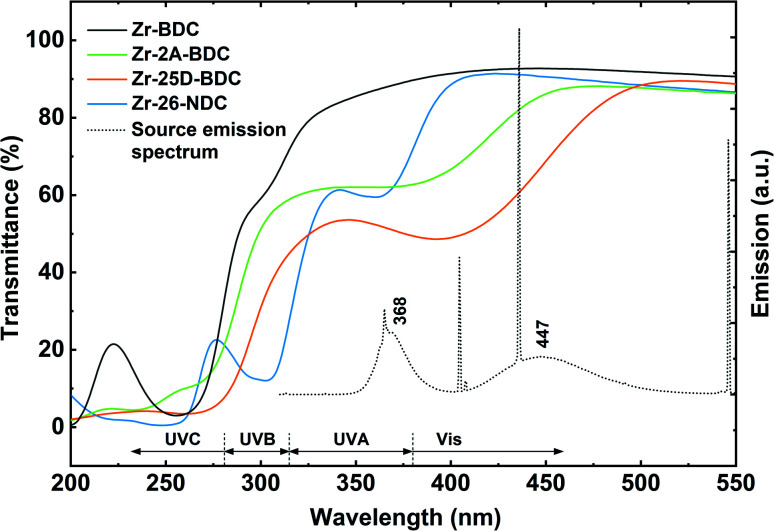
UV-Vis transmission spectra of Zr-hybrids with different aromatic linkers: Zr-BDC (130 nm), Zr-2A-BDC (108 nm), Zr-25D-BDC (116 nm) and Zr-26-NDC (109 nm). The dotted line shows the emission spectrum of the light source used in the bacteria inactivation experiments. The Zr-BDC film (ZrCl_4_ + 1,4-benzenedicarboxylic acid) was deposited as described in the literature,^25^ with the standard pulsing sequence of 4–2–3–1 s at 265 °C.

The optical properties of the Zr-25D-BDC film were further compared with systems based on alternative aromatic dicarboxylic acids, *i.e.* Zr-BDC (1,4-benzenedicarbozylic acid), Zr-2A-BDC (2-amino-1,4-benzenedicarboxylic acid) and Zr-26-NDC (2,6-naphthalenedicarboxylic acid). The possible structures of the Zr-organic hybrid films evaluated in this work are presented in Fig. 6. The UV-Vis transmission spectra of the Zr-organic hybrid films show that the position of characteristic π–π* transition for aromatic rings strongly depends on the choice of the organic component in the hybrid film (Fig. 7). The Zr-BDC film is fully transparent in the visible and near-UV region, showing the absorption onset at around 315 nm. The presence of amino and hydroxyl groups in the aromatic linker alters the absorption properties of the films. These functional groups influence the conjugated system of 1,4-benzenedicarbozylic acid and cause a shift of the absorption peaks towards longer wavelengths. This is particularly evident when two OH groups are present. The Zr-2A-BDC system also shows absorption in the visible region, with the onset around 450 nm. In contrast, the naphthalene ligand with an extended conjugated system causes much weaker absorption in the visible region with the onset around 400 nm and *λ*_max_ = 360 nm. Most importantly, the presence of the intense absorption in the UVA and visible region of the complexes suggests that these materials can be potentially used in antimicrobial photodynamic therapy, depending on the optical penetration depth required for various treatments.

### Wetting characteristics

Wetting properties of the surfaces can influence antibacterial behaviour and cell attachments to a certain degree, but its measured value can often be precluded by a slowly dissolving film.^26,27^ The effect of water on surface properties was studied by submerging the films in distilled water for 3 h (Table 1), the same duration as required for antibacterial assays and bacteria adhesion to the surfaces. The thickness of the films slightly decreases after 3 h of water treatment of about 6% for the Zr-26-NDC, 3% for the Zr-2A-BDC, and 1% for the Zr-25D-BDC film. This reduction is rather low and proves that the films are sufficiently stable in aqueous solutions. Furthermore, no visible changes were observed on the film surfaces after water treatment.

**Table 1:** Film thickness before and after 3 h of exposure to DI water as measured by spectroscopic ellipsometry

Sample	Initial thickness (nm)	Thickness after 3 h (nm)
Zr-26-NDC	110.2	103.7
Zr-2A-BDC	120.8	117.0
Zr-25D-BDC	113.3	111.9

**Fig. 8:**
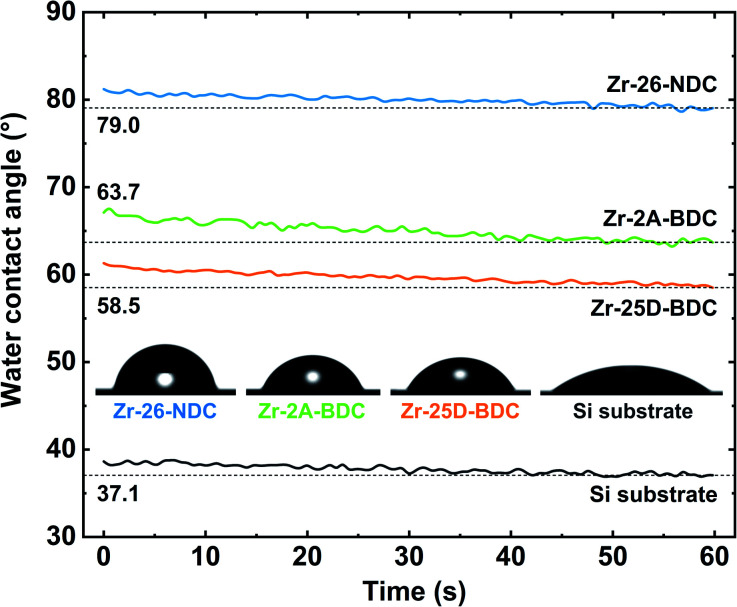
Contact angle between water and films: Zr-26-NDC (79°), Zr-2A-BDC (63°), Zr-25D-BDC (58°) and clean Si substrate (37°). The horizontal lines show the final value of the contact angle in each case.

The wettability of the Zr-25D-BDC, Zr-2A-BDC and Zr-26-NDC films was further investigated by measuring the contact angle of water on the surfaces (Fig. 8). The wetting on a silicon substrate with a native oxide layer was measured for comparison. All films exhibit a rather hydrophilic nature, with contact angles of 58.5°, 63.7° and 79.0° for Zr-25D-BDC, Zr-2A-BDC and Zr-26-NDC, respectively. The wetting of the Zr-26-NDC system appears to be more shifted towards a hydrophobic character as compared to the other systems. This indicates that the surface wettability depends on the surface chemistry and organic component in the hybrid films. This can be related to different polarizability of the functional groups and thus the surface free energy, but also to the size of the conjugated system. The long 26-NDC linker decreases the wettability of the surface (higher contact angle). Furthermore, the presence of –NH_2_, and –OH groups can increase the polarizability and thus its wettability,^28,29^ which is consistent with the measured contact angles for Zr-2A-BDC and Zr-25D-BDC. Additionally, we show an evolution of the contact angle *versus* time. The contact angle for each surface exhibits a rather small change of about 2.4° and reaches equilibrium after around 45 s, supporting that the films are stable in water solution.

### Antibacterial properties

**Fig. 9:**
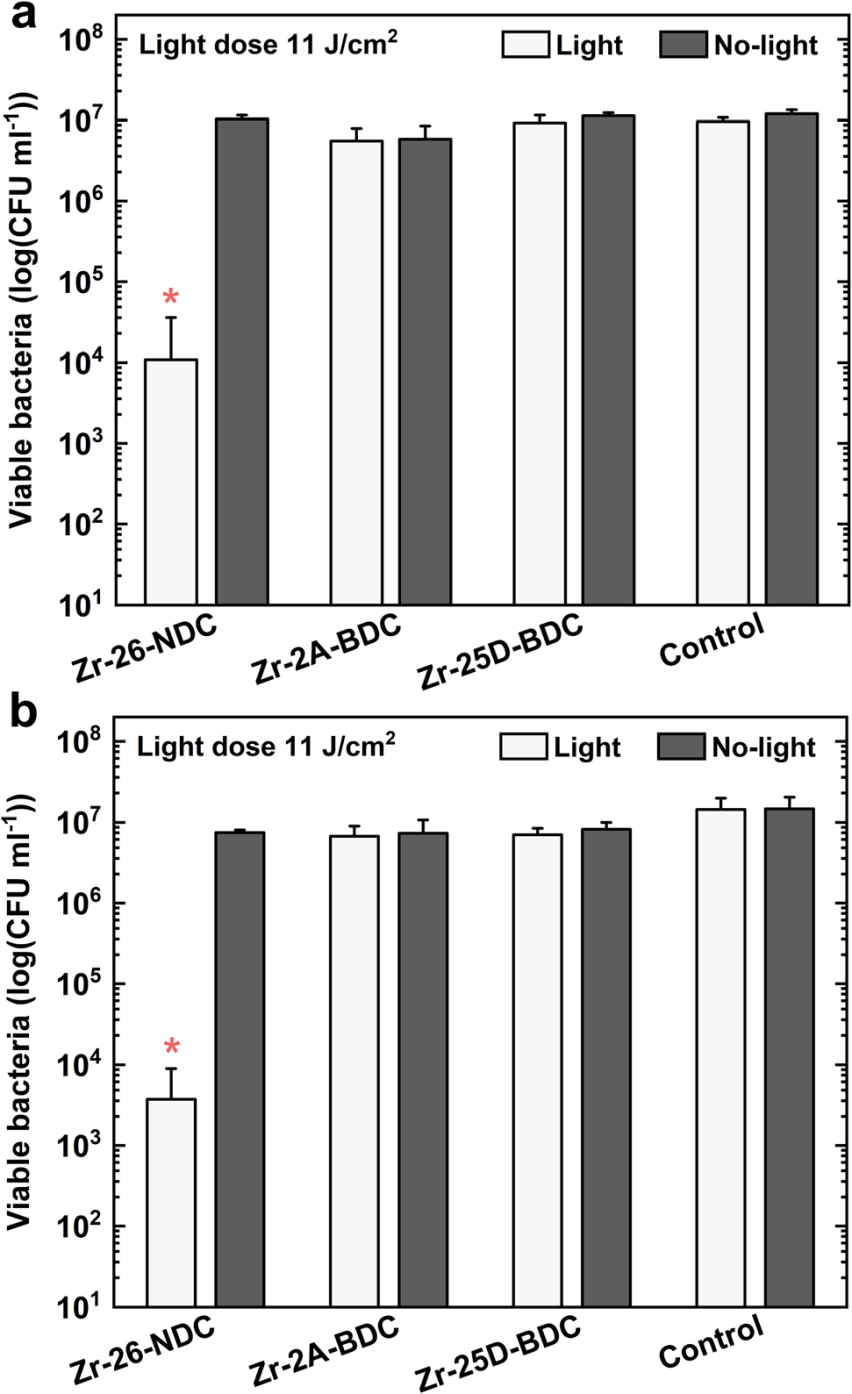
Phototoxic effect against *S. aureus* in planktonic form after exposure to hybrid films, including Zr-26-NDC (*n* = 9), Zr-2A-BDC (*n* = 6), Zr-25D-BDC (*n* = 6) and uncoated coverslips as controls (*n* = 6) combined with UVA/blue light irradiation (11 J cm^−2^). (a) Attached bacteria after 90 min of incubation, and (b) free-floating bacteria in the supernatant above the film from the same experiment. A statistically significant difference from all other samples is marked by a red asterisk, *p* < 0.05.

The bacterial phototoxicity of photoactive Zr-2A-BDC, Zr-25D-BDC and Zr-26-NDC samples with relatively similar thicknesses of about 110 nm was tested against *S. aureus* in planktonic form. The number of viable bacteria was measured both for surface-attached bacteria after 90 min of incubation (Fig. 9a) and for free-floating bacteria in the supernatant above the film surface from the same experiment (Fig. 9b). The phototoxicity studies were performed using a combination of UVA and blue light irradiation sources with the emission that overlaps to a great extent with the absorption spectra of all tested films, see Fig. 7. In addition, the hybrid thin films show high stability under UVA and blue light irradiation (20 min) as proved by the UV-Vis measurements and thickness analysis by spectroscopic ellipsometry (data not shown).

The irradiated Zr-26-NDC surface reached around 99.91% reduction of the attached bacteria and 99.97% reduction of free-floating bacteria in the solution above the surface as compared to non-irradiated control samples. Surprisingly, no significant reduction was observed for Zr-25D-BDC and Zr-2A-BDC samples both for surface-attached and free-floating bacteria. This indicates that the longer 26-NDC molecule with a larger conjugated system has superior antibacterial activity than the shorter, one ring 2A-BDC and 25D-BDC molecules, even though the absorption is brought into the visible range. Two possible factors may impact the PDT efficacy, the reactive oxygen species quantum yield and/or type of generated species that enters the antibacterial process.^30,31^ ROS can be generated *via* type I (superoxide anion radical O_2_˙^−^, hydrogen peroxide H_2_O_2_ or hydroxyl radical HO˙) or type II (singlet oxygen ^1^O_2_) reaction mechanism, whereas most photosensitizers used in PDT are believed to operate *via* type II rather than type I mechanism.^2^ We have not identified the type of generated ROS in our Zr-26-NDC system, but we can assume the formation is mostly of singlet oxygen, which is generally considered as the primary damaging species in PDT.^32^ This implies a higher ^1^O_2_ quantum yield, which means the higher number of singlet oxygen molecules produced per absorbed photon for the Zr-26-NDC system as compared to the Zr-2A-BDC and Zr-25D-BDC systems. On the other hand, the photosensitization process in Zr-26-NDC system may also involve mixed type I and type II reactions with formation of highly reactive hydroxyl radicals. To pinpoint these mechanisms remains for further studies.

It is also worthwhile noting that not only the size of the conjugated system but also the type of the functional group and the polarity characteristic of the ligand can impact interaction and attachment of the molecule to the Gram-positive bacteria, and thus the antibacterial activity of the created systems. Moreover, the 2A-BDC and 25D-BDC light-absorbing molecules can be ineffective photosensitizers in PDT due to the possible internal conversion and radiationless decay of these molecules to the ground state without emitting a photon.

Another possible explanation for lack of phototoxic activity of the Zr-2A-BDC and Zr-25D-BDC films is the level of overlap between the light absorbance of these films and emission from the lamp that can affect the ROS quantum yield (Fig. 7). The Zr-NDC film overlaps with the irradiation source only in the UVA region, while the Zr-2A-BDC and Zr-25D-BDC films both in the UVA and visible part of the spectrum. However, no phototoxicity of the Zr-25D-BDC complex was observed when a different light source configuration was used consisting of two side blue light tubes and one middle UVA tube giving the average irradiance of 12 mW cm^−2^ (±5%) (data not shown), with relatively similar emission spectrum. The light dose of this configuration was higher than in the previous configuration giving about 14 J cm^−2^ after 20 min of irradiation. Increasing the irradiation time and thus radiant exposure even higher could potentially increase the phototoxic effect of these samples. Importantly, no significant antibacterial effect was observed for the samples without light irradiation (dark toxicity), pointing to high stability and apparent lack of toxicity of the created thin films.

The antibacterial activity of our Zr-26-NDC sample was higher than those reported by Lausund *et al.*,^11^ where they show about 50% reduction of viable bacteria. It should be noted, however, that in the present study a different bacterial species and a different UVA light source were used.

**Fig. 10:**
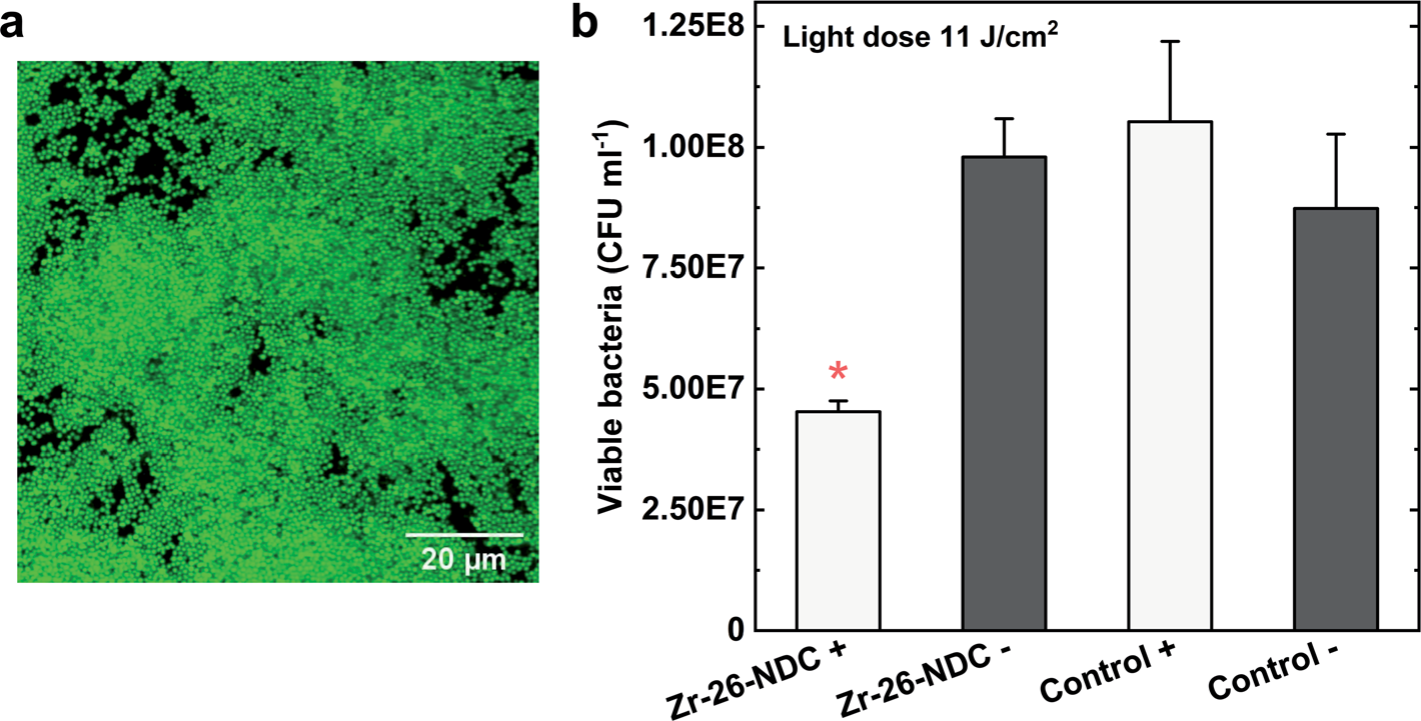
(a) Confocal laser scanning microscopy image of *S. aureus* biofilm grown for 24 h on Zr-26-NDC film. Biofilm was stained with DNA-binding dye SYTO 9 and cells are green. (b) Phototoxic effect against *S. aureus* biofilm after exposure to Zr-26-NDC film (*n* = 6) and uncoated coverslips as controls (*n* = 4) combined with (+) UVA/blue light irradiation (11 J cm^−2^). A statistically significant difference from all other samples is marked by a red asterisk, *p* < 0.05.

The antibacterial activity of the Zr-26-NDC sample was further investigated against *S. aureus* bacteria in a biofilm. The formation of a 24 h biofilm on the surface was first verified with confocal laser scanning microscopy (CLSM) (Fig. 10a). The CLSM images confirmed the formation of a relatively dense biofilm on the film surface. The Zr-26-NDC surface in combination with UVA/blue light irradiation (11 J cm^−2^) was effective against established biofilm (*p* < 0.05) (Fig. 10b). Around 57% reduction of viable bacteria was observed for irradiated Zr-26-NDC (+) sample as compared to the irradiated controls, and around 48% reduction as compared to the non-irradiated controls. Increasing the irradiation dose to about 16 J cm^−2^ (30 min of irradiation) could potentially increase the phototoxic effect against created biofilm. Moreover, no reduction of viable bacteria was observed for non-irradiated Zr-26-NDC (−) samples, which proves lack of cytotoxicity and high stability of this system in bacteria solution.

## Conclusions

In this work, we have developed a new MLD process for fabricating photoactive hybrid thin films based on Zr clusters and aromatic 25D-BDC organic linkers. The amorphous and uniform films display high absorption in the visible part of the spectrum. Moreover, we show that the type of the organic ligand and its functional groups can be used to tune the optical properties of Zr-organic hybrids, as shown by the Zr-2A-BDC and Zr-26-NDC systems in this work. The presence of amino –NH_2_ and especially hydroxyl –OH functional groups on the aromatic linker broadens and shifts the optical absorption of the films towards the visible part of the spectrum. Our results also indicate that the type of the organic ligand changes the wetting properties of the surfaces. All films are hydrophilic, while the Zr-26-NDC film is more towards a hydrophobic nature. The antibacterial properties of the films are dependent on the type of ligand used. The phototoxicity experiments against *S. aureus* Gram-positive bacteria reveals a lack of activity for the Zr-25D-BDC and Zr-2A-BDC systems, whereas a high activity is observed for the Zr-26-NDC systems, both against planktonic bacteria and bacteria in biofilms. Antibacterial activity, absence of dark toxicity and high stability in contact with water of Zr-26-NDC system indicates potential for application of this hybrid material in photodynamic therapy.

## Conflicts of interest

There are no conflicts to declare.

## Supplementary Material
